# Effects of a Nanoparticle-Loaded PVA/SPI Pad on Microbial Proliferation and Quality Characteristics of Superchilled Pork

**DOI:** 10.3390/foods15162830

**Published:** 2026-08-14

**Authors:** Haoyue Wu, Yi Zhou, Huaxing Xu, Zhaoming Wang, Xingguang Chen, Hui Zhou

**Affiliations:** 1 Key Laboratory for Animal Food Green Manufacturing and Resource Mining of Anhui Province, Hefei University of Technology, Hefei 230601, China; 18730099801@163.com (H.W.);; 2 Engineering Research Center of Bio-Process, Ministry of Education, Hefei University of Technology, Hefei 230601, China; 3 School of Food and Biological Engineering, Hefei University of Technology, Hefei 230601, China

**Keywords:** 16S rRNA gene sequencing, active packaging, microbial proliferation, pork spoilage, superchilled storage, water-holding capacity

## Abstract

Fresh pork remains susceptible to psychrotrophic spoilage during superchilled storage. Although oregano essential oil (OEO), nisin, and active absorbent pads have each been studied, their effects on spoilage-community proliferation and concurrent quality loss remain poorly resolved. Here, we coupled longitudinal 16S rRNA gene profiling with conventional microbiological and quality measurements to evaluate a poly(vinyl alcohol)/soy protein isolate pad containing OEO-loaded soluble soybean polysaccharide-nisin nanoparticles (PS-NPs). Pork was stored at −1 °C for 24 days without a pad (CK), with a nanoparticle-free pad (PS), or with PS-NPs. Bacterial communities in CK and PS-NPs were profiled alongside total viable count, TVB-N, TBARS, protein carbonyls, color, water-holding capacity, and sensory quality. Compared with CK, PS-NPs slowed the increase in viable counts and delayed physicochemical and sensory deterioration. On day 24, *Pseudomonas* relative abundance was 39.48% with PS-NPs and 51.02% in CK. TBARS and protein carbonyl contents were 36.96% and 19.44% lower than CK, respectively, while cooking loss was 28.49% versus 32.18%. Among the evaluated taxon-quality pairs, *Pseudomonas psychrophila* showed the strongest positive association with protein carbonyl content. Unlike earlier performance-focused studies, this work links packaging-associated community shifts with concurrent chemical, physical, and sensory changes under superchilling. The findings support PS-NPs as a preservation strategy.

## 1. Introduction

Fresh pork is highly perishable because its high water activity (a_w_ ≈ 0.99), near-neutral pH, and abundant nutrients support rapid microbial growth [1]. In modern food industry practices, cold-chain logistics—particularly superchilled storage—are widely used to delay quality deterioration [2]. However, psychrotrophic bacteria remain metabolically active under these conditions and can promote oxidative deterioration and off-odor formation. Additional preservation strategies, including active packaging, are therefore required to complement temperature control [3,4]. In active packaging, incorporated agents act within the food-package system to slow spoilage and preserve product quality [5,6,7,8]. Active packaging can inhibit microbial growth, limit oxidation, or absorb meat exudates, depending on its incorporated components [9]. Accordingly, active packaging has received increasing attention as a preservation strategy for chilled pork [10,11]. Among various active packaging systems used for meat preservation, active absorbent pads represent a commonly applied form [12,13].

The present study builds on a previously developed nanoparticle-loaded active pad [14]. Free OEO is poorly dispersible in water, volatile, and susceptible to oxidation, while nisin activity can be limited by interactions with food components. Encapsulation is therefore expected to protect OEO, improve its dispersion in the aqueous pad matrix, and moderate release at the meat–package interface [14,15]. OEO phenolics can disrupt bacterial membrane integrity and homeostasis. Nisin binds lipid II, inhibits cell-wall biosynthesis, and forms membrane pores in susceptible bacteria [16]. Combining these agents in ONSNPs may provide complementary membrane activity and sustained local delivery. These benefits are mechanistic expectations, because the present storage experiment did not include free-OEO or free-nisin comparison groups.

In untreated pork, superchilling slows but does not stop deterioration, and recent control-focused studies show that the trajectory depends strongly on temperature and ice formation. Across −1 to −3 °C, control pork exhibited progressive increases in total aerobic count and TVB-N, together with lipid and protein oxidation; deeper superchilling slowed these reactions but could increase cooking loss when ice crystals disrupted muscle structure [17]. Under a constant superchilling temperature, these changes occurred more slowly, whereas temperature fluctuations accelerated TVC, TVB-N, TBARS formation, muscle-fiber disruption, free-water migration, and drip loss [18,19]. In realistic cold-chain simulations at −1 °C, quality loss also coincided with microbial proliferation and restructuring of the dominant community, confirming that temperature control alone cannot fully suppress spoilage [20]. These untreated-control patterns identify a need for a complementary intervention that simultaneously limits microbial growth, oxidation, and exudate accumulation. A poly(vinyl alcohol)/soy protein isolate pad containing nanoparticles (PS-NPs) is promising because it combines moisture management with localized delivery of OEO and nisin. The closest existing study developed this PS-NPs pad and demonstrated its structure, release behavior, and preservation performance at 4 °C [14], but it did not determine how the pad relates to microbial proliferation and multiple quality trajectories during superchilling; the present work addresses that unresolved connection.

Previous work established the structure, controlled-release behavior, and preservation performance of this pad at 4 °C [14]. However, it did not resolve temporal bacterial proliferation or connect community shifts with nitrogenous spoilage, lipid and protein oxidation, water-holding capacity, and sensory decline under superchilling. Here, pork was stored at −1 °C with CK, PS, or PS-NPs treatments. We combined 16S rRNA gene sequencing of CK and PS-NPs samples with TVC, TVB-N, TBARS, protein carbonyl, color, water-holding, and sensory measurements. We then tested associations between dominant bacterial taxa and quality indices to determine whether packaging-associated community changes coincided with delayed deterioration.

## 2. Materials and Methods

### 2.1. Schematic Overview of Experimental Program

**Figure 1:**
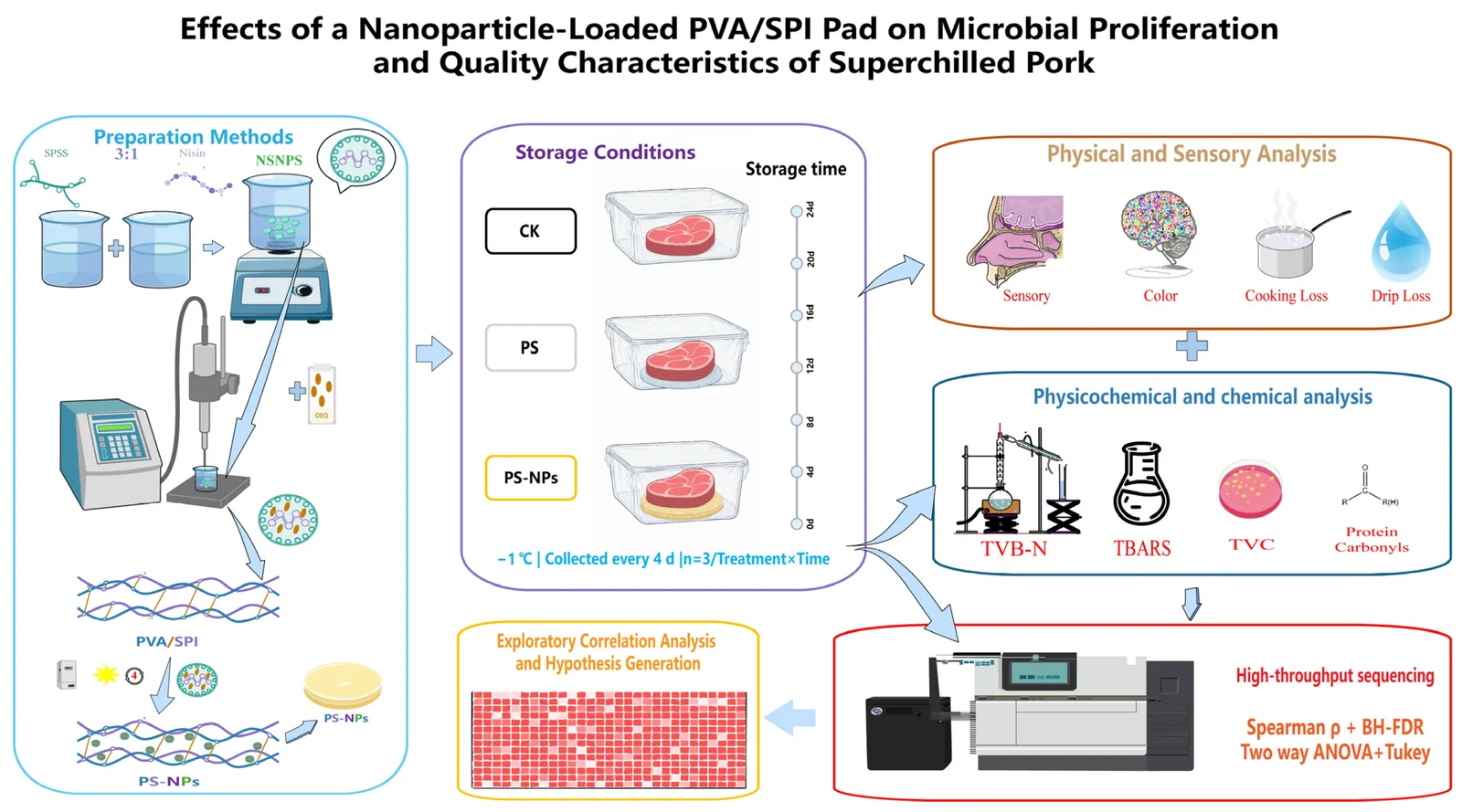
Schematic overview of the experimental program, from PS-NPs preparation and pork treatment through superchilled storage, quality analyses, and integrated statistical interpretation.

Figure 1 summarizes the preparation of the active pad, allocation of pork to CK, PS, and PS-NPs treatments, superchilled storage, scheduled sampling, analytical measurements, and data integration. The workflow was designed to test whether PS-NPs altered microbial proliferation and delayed chemical, physical, and sensory deterioration during storage at −1 °C. All experimental procedures were conducted according to good laboratory practices in the Key Laboratory for Animal Food Green Manufacturing and Resource Mining of Anhui Province, Hefei University of Technology. Authenticity and validity were supported through independent biological replicates, defined control groups, consistent sample handling and sampling times, method-specific blanks or calibration standards where applicable, and prespecified statistical analyses.

### 2.2. Materials and Reagents

Plate Count Agar (PCA), analytical grade, was purchased from Guangdong Huankai Microbial Technology Co., Ltd. (Guangzhou, China). Boric acid and magnesium oxide (analytical grade) were obtained from Shanghai Macklin Biochemical Co., Ltd. (Shanghai, China). Methyl red, bromocresol green, and thiobarbituric acid (TBA) (analytical grade) were supplied by Aladdin Reagent (Shanghai, China) Co., Ltd. (Shanghai, China). Trichloroacetic acid (TCA), sodium chloride, and disodium ethylenediaminetetraacetate (EDTA-2Na) (analytical grade) were acquired from Sinopharm Chemical Reagent Co., Ltd. (Shanghai, China). Total sulfhydryl content kits (Cat. No. BC1270) were purchased from Beijing Solarbio Science & Technology Co., Ltd. (Beijing, China).

### 2.3. Sample Preparation

#### 2.3.1. Preparation of Ternary Nanoparticles

Ternary nanoparticles were produced using a modified version of the procedure described by Luo et al. [21]. Soluble soybean polysaccharide (food grade, Tianjin Fuji Protein Co., Ltd., Tianjin, China) was dissolved in ultrapure water at 3 mg/mL by magnetic stirring (Model DF-101S, Shanghai Hujia Instrument Equipment Co., Ltd., Shanghai, China) for 120 min. A separate 3 mg/mL nisin (analytical grade, Macklin Biochemical Co., Ltd., Shanghai, China) solution was prepared in 1% (*v*/*v*) acetic acid (analytical grade, Aladdin Reagents (Shanghai) Co., Ltd., Shanghai, China) and added slowly at an SSPS-to-nisin ratio of 3:1. After 60 min of stirring, the mixture was centrifuged at 3500 rpm for 10 min. The supernatant was passed through a 0.45 μm membrane (Jinteng Experimental Equipment Co., Ltd., Tianjin, China) to yield NSNPs. Oregano essential oil (analytical grade, Shanghai Yuanye Bio-Technology Co., Ltd., Shanghai, China) was added dropwise to the NSNP suspension at 1%, 2%, 3%, 4%, or 5% (*v*/*v*). Each formulation was stirred at 500 rpm for 30 min and sonicated at a nominal power of 350 W for 10 min using 2 s pulses separated by 3 s intervals. Sonication was performed using an ice bath for temperature control with a SCIENTZ-IID ultrasonicator (Ningbo Scientz Biotechnology Co., Ltd., Ningbo, China), fitted with a 6 mm probe at 350 W output power and 100% amplitude. The sonication was conducted in pulsed mode for a total duration of 10 min (2 s on, 3 s off). The actual energy input was not recorded. The resulting formulations were designated ONSNPs-I through ONSNPs-V in order of increasing oil concentration [15].

#### 2.3.2. Preparation of PS-NPs Active Pads

PS-NPs pads were fabricated by dispersing oregano essential oil-loaded soluble soybean polysaccharide-nisin nanoparticles (ONSNPs) in a PVA/SPI matrix. We followed the procedure of Xu et al. [14], which was based on the freeze-thaw approach reported by Varshney et al. [22]. PVA (7 g) (analytical grade, Macklin Biochemical Co., Ltd., Shanghai, China) was dissolved in 100 mL ultrapure water under magnetic stirring at 95 °C for 1 h. After a clear viscous solution formed, 3 g SPI (analytical grade, Shanghai Yuanye Bio-Technology Co., Ltd., Shanghai, China) powder was incorporated and stirred at 60 °C for another hour. The final PVA/SPI precursor contained 10% (*w*/*v*) total polymer. ONSNPs were mixed into this precursor at 15% (*v*/*v*), followed by mechanical stirring for 1 h. A 25 g aliquot of the pad-forming mixture was poured into a 90 mm plastic Petri dish (Biosharp, Lanjieke Technology Co., Ltd., Beijing, China). Each specimen underwent four freeze–thaw cycles, comprising 12 h at −20 °C and 12 h at room temperature per cycle. Following the fourth thaw, the pads were dried for 12 h at 37 °C to obtain the final PS-NPs material [15].

#### 2.3.3. Treatment of Fresh Pork

Chilled pork longissimus thoracis (LT; commonly referred to as longissimus dorsi) was purchased from an RT-Mart supermarket in Hefei, China and transported to the laboratory under refrigerated conditions. The meat originated from commercial crossbred finishing pigs slaughtered at approximately 170 days of age. The postmortem interval at sample preparation was approximately 36 h. According to the supplier records, the initial pH measured at 45 min postmortem and the ultimate pH measured at 24 h postmortem were approximately 6.25 and 5.60, respectively. The mean pH measured in the pork samples at the beginning of the experiment (day 0) was 5.70. Fascia, visible adipose tissue, and connective tissue were aseptically removed before the pork was divided among three treatment groups. The control group (CK) contained no absorbent pad, whereas the PS group contained a nanoparticle-free PVA/SPI pad. The PS-NPs group contained a PVA/SPI pad incorporated with 15% (*v*/*v*) ONSNPs. Each tray was wrapped with plastic film and stored at −1 °C. Samples were analyzed at 4-day intervals until day 24.

### 2.4. Microbiological Analysis

#### High-Throughput Sequencing

Three independent biological replicates were sequenced for CK on days 0, 8, 16, and 24 and for PS-NPs on days 8, 16, and 24, with the day-0 CK samples serving as the common baseline (*n* = 21 samples in the reported community and correlation analyses). Majorbio Bio-Pharm Technology Co., Ltd. (Shanghai, China) completed library construction and sequencing. Genomic DNA was isolated with the FastPure Soil DNA Isolation Kit (Majorbio (Shanghai Majorbio Bio-pharm Technology Co., Ltd.), Shanghai, China). The bacterial V3-V4 region was amplified using the primers 338F and 806R. The amplicons were purified with an AxyPrep DNA Gel Extraction Kit (Axygen Biosciences (Corning Life Sciences), Union City, CA, USA). A Quantus™ fluorometer (Illumina, Inc. (Majorbio Bio-pharm Technology Co., Ltd.), San Diego, CA, USA) was used for product quantification. Paired-end reads were generated on an Illumina MiSeq PE300 platform according to the provider’s protocol. Raw reads were filtered with Fastp v0.19.6 and joined with FLASH v1.2.11. UPARSE v7.1 clustered sequences into OTUs at 97% similarity, after which chimeras were discarded. Taxonomy was assigned with RDP Classifier v11.5 against SILVA at a confidence cutoff of 0.7 [20,23]. Prior to downstream diversity analyses, the OTU abundance table was normalized by equal-depth subsampling to 40,137 sequences per sample. Alpha-diversity indices, including Sobs, Chao1, ACE, Shannon, Simpson, and Good’s coverage, were calculated from OTUs clustered at 97% sequence similarity using Mothur v1.30.2. The Simpson index is reported in its dominance form (D), with larger values indicating greater community dominance. The random seed used for subsampling was not recorded in the archived analysis outputs. Beta diversity was evaluated using Bray–Curtis dissimilarities calculated from the rarefied 97% OTU abundance table, with 40,137 sequences retained per sample. The analysis included 21 samples: CK samples collected on days 0, 8, 16, and 24 and PS-NPs samples collected on days 8, 16, and 24. The three CK samples collected on day 0 were included once as the common baseline. PCoA was performed at the OTU level, and differences in overall community composition among the seven observed treatment–time groups were evaluated using PERMANOVA on the Majorbio Cloud Platform. The homogeneity of multivariate dispersion was assessed using PERMDISP based on Bray–Curtis distances to group spatial medians. Statistical significance was evaluated using 9999 permutations with a fixed random seed of 20260804. PERMDISP was performed for the seven-group model based on all 21 samples and separately for the Treatment and Time groupings based on the 18 post-storage samples.

### 2.5. Physicochemical and Chemical Analysis

#### 2.5.1. Total Volatile Basic Nitrogen (TVB-N)

TVB-N was determined using an automatic Kjeldahl nitrogen analyzer following a semi-micro method adapted from Ning et al. [24]. Briefly, 10.0 g of minced pork was mixed with 75 mL of deionized water and extracted for 30 min. After Kjeldahl distillation, the released volatile bases were quantified by standardized HCl titration against a reagent blank. The aqueous extract was transferred to a K9860 Kjeldahl analyzer (Hanon Instruments Co., Ltd., Jinan, China) for distillation. Magnesium oxide (1 g) was added to release volatile basic compounds and the distillate was collected in 25 mL of a 20 g L^−1^ boric acid solution containing a mixed methyl red–bromocresol green indicator. The trapped volatile bases were titrated with standardized 0.0100 mol L^−1^ hydrochloric acid, with a reagent blank processed in parallel. The titration endpoint was identified by a color change from blue-green to faint reddish-pink. The results were expressed as milligrams of nitrogen per 100 g of pork. TVB-N was calculated as:

(1) $$X=\frac{(V_{1}-V_{2})\times c\times 14}{m}\times 100$$

where (*V*_1_) and (*V*_2_) are the HCl volumes consumed by the sample and blank, respectively, (*c*) is the HCl concentration, and (*m*) is the sample mass.

#### 2.5.2. Total Viable Counts of Bacteria (TVC)

TVC was measured by a plate-count procedure adapted from Zhou et al. [25]. A 10 g minced-pork portion was combined with 90 mL sterile saline in a sterile homogenization bag and processed for 2 min. The homogenate was serially diluted tenfold and 1 mL of a suitable dilution was spread on PCA. Colonies were enumerated after incubation at 37 °C for 48 h. Each treatment-by-time combination included three independent biological replicates.

#### 2.5.3. Thiobarbituric Acid Reactive Substances (TBARS)

TBARS were analyzed using a modified GB 5009.181-2016 procedure [26]. Minced pork (3 g) was homogenized with 30 mL trichloroacetic acid solution. After shaking at 50 °C for 30 min, the mixture was cooled to room temperature and filtered. An equal volume of thiobarbituric acid solution was combined with the filtrate. The reaction mixture was held in a 90 °C water bath for 30 min and then cooled. Absorbance was recorded at 532 nm. Calibration used 1,1,3,3-tetraethoxypropane standards from 0.125 to 1.5 mg/kg. TBARS were calculated from the resulting MDA concentration and reported as mg MDA/kg. Sample and reagent blank absorbances were processed against the same calibration curve, and the measured malondialdehyde equivalent was normalized to the initial meat mass.

#### 2.5.4. Determination of Protein Carbonyls

Protein carbonyl content was measured with a commercial 2,4-dinitrophenylhydrazine (DNPH) assay kit (BC1270; Beijing Solarbio Science & Technology Co., Ltd., Beijing, China). Approximately 0.1 g of tissue was homogenized in 1.0 mL kit extraction buffer and centrifuged at 4 °C and 5000 rpm for 10 min. The recovered protein fraction was reacted with DNPH while a matched reagent blank was processed in parallel. After protein precipitation and washing to remove unreacted DNPH, the derivatized protein was dissolved according to the kit protocol and absorbance was read at 370 nm. Carbonyl concentration was calculated using the kit equation and normalized to protein content, with results expressed as nmol carbonyl/mg protein.

### 2.6. Physical and Sensory Analyses

#### 2.6.1. Sensory Evaluation

Ten trained panelists independently evaluated the color, odor, and overall acceptability of the pork samples at each designated storage time. Samples were identified using coded labels and presented in a randomized order [27]. Panelists were blinded to treatment identity, and the presentation sequence was independently randomized for each panelist to minimize identification and order biases. Evaluations were based exclusively on visual observation and orthonasal odor assessment; tasting, ingestion, and direct contact with the samples were prohibited. Each biological replicate was evaluated by all panelists, and the mean panel score for each attribute was used as the sensory value of that biological replicate. Individual ratings were recorded using study codes and analyzed in anonymized, aggregate form. The protocol was approved by the Biomedical Ethics Committee of Hefei University of Technology (approval NO. HFUT20260127001H; 27 January 2026). All panelists were informed of the procedures and provided written informed consent before participation.

#### 2.6.2. Evaluation of Color Change

Pork color was assessed with a portable spectrophotometer following Chatkitanan et al. [28], and L*, a*, and b* were recorded for each specimen. Instrumental color was measured using a TS7600 spectrophotometer (3nh Technology Co., Ltd., Shenzhen, China). The instrument was operated using the CIE standard illuminant D65, a 2° standard observer and an 8-mm measurement aperture. Before each measurement session, the spectrophotometer was calibrated using the manufacturer-supplied white and black calibration standards. CIE L*, a*, and b* values were recorded at three non-overlapping positions on the exposed muscle surface. The three readings were averaged to obtain a single color value for each biological replicate.

#### 2.6.3. Drip Loss

Drip loss was evaluated using the protocol of Deng et al. [29]. Each sample was weighed before storage to obtain *m*_1_. At sampling, exuded surface liquid was removed with sterile filter paper before reweighing the sample as *m*_2_. Drip loss was calculated using the formula below. The same balance was used for initial and final weighing and samples were handled for a consistent interval before the final mass was recorded. Values were normalized to the initial mass and expressed as a percentage for each independent biological replicate.

(2) $$\mathrm{Drip}\;\mathrm{Loss}=\frac{\left(m_{1}-m_{2}\right)}{m_{1}}\times 100\%$$

#### 2.6.4. Cooking Loss

Pork was portioned into 4 cm × 4 cm pieces and weighed to obtain *m*_3_. The portions were vacuum-sealed in cooking bags and heated at 71 °C for 35 min. They were subsequently cooled under running water for 30 min. After removing surface liquid with filter paper, each portion was weighed again as *m*_4_ [30]. Cooking loss was calculated using the formula below. The sealed portions were fully immersed during heating, and the same cooling and blotting procedure was applied to all groups before final weighing. Cooking loss was calculated as 100 × (*m*_3_ − *m*_4_)/*m*_3_ for each independent biological replicate.

(3) $$\mathrm{Cooking}\;\mathrm{Loss}=\frac{\left(m_{3}-m_{4}\right)}{m_{3}}\times 100\%$$

### 2.7. Statistical Analysis

At each post-treatment sampling time, three biological replicate values were analyzed per treatment. The three day-0 samples constituted a shared pretreatment baseline rather than independent treatment-specific replicates. Within-sample color readings and panelist scores were averaged to obtain one value per biological replicate before statistical analysis. These within-sample measurements were not treated as independent observations. Data are reported as mean ± standard deviation.

Statistical analyses were performed using SPSS 26.0, and figures were prepared using Origin 2021. Each outcome was analyzed separately using two-way ANOVA to evaluate the effects of pad treatment, storage duration, and their interaction. Post hoc pairwise comparisons were performed using Tukey’s honestly significant difference test within each outcome. This adjustment did not control multiplicity across different physicochemical and sensory outcomes. Exploratory Spearman rank correlations were calculated between dominant-taxon relative abundances and matched physicochemical or sensory measurements from 21 samples. The three day-0 samples were included once as the shared baseline in this correlation dataset. The Benjamini–Hochberg procedure was applied across all taxon–quality correlation tests, with adjusted *p* values < 0.05 considered significant. Hierarchical clustering was used to order the rows and columns of the correlation heatmap.

## 3. Results and Discussion

### 3.1. Bacterial Community Proliferation and Diversity in Pork During Storage

#### 3.1.1. Alpha Diversity Analysis of Bacterial Communities

**Figure 2:**
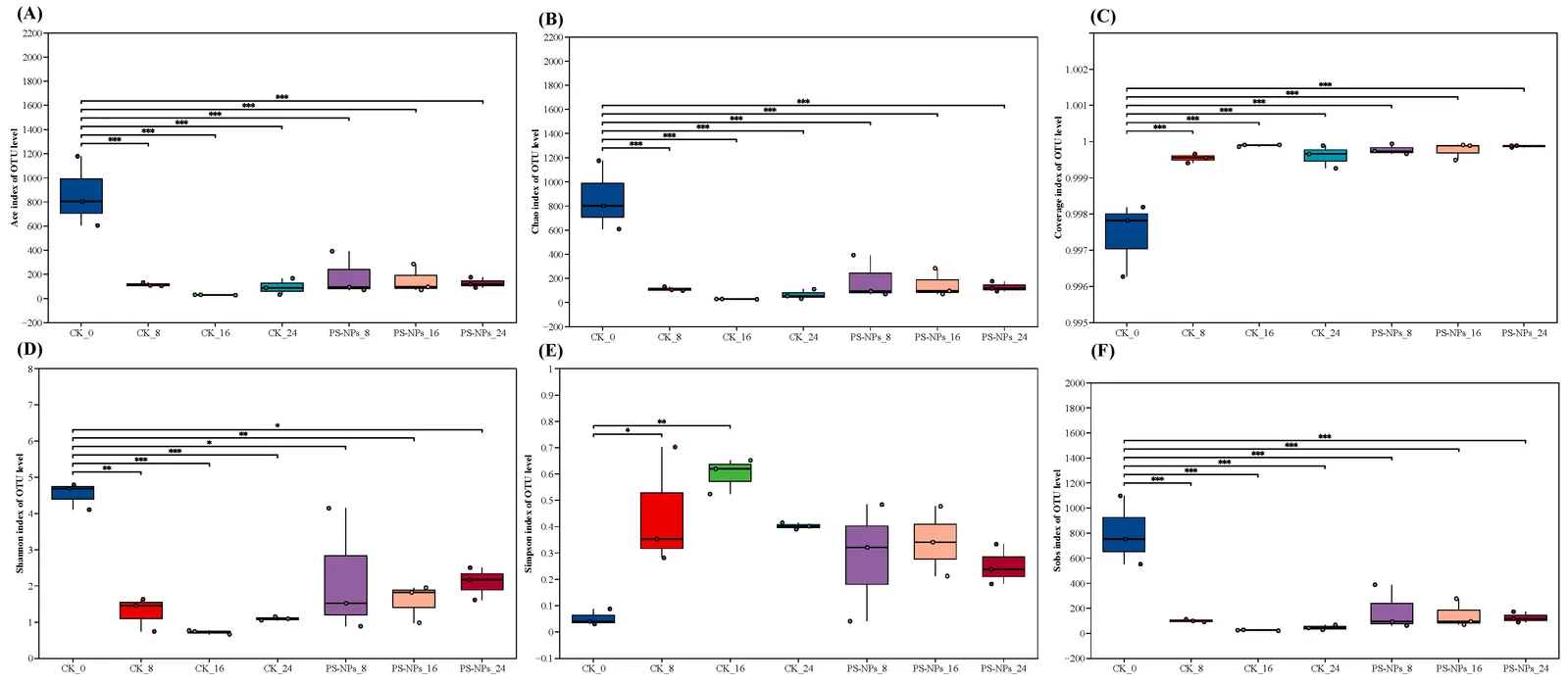
Alpha-diversity metrics of pork microbiota across storage treatments: (**A**) ACE, (**B**) Chao 1, (**C**) Coverage, (**D**) Shannon, (**E**) Simpson, and (**F**) Sobs. Asterisks denote statistically significant differences between compared groups. * *p* < 0.05, ** *p* < 0.01, *** *p* < 0.001.

The 21 samples included in the community and correlation analyses yielded 1,331,274 quality-filtered and merged reads comprising 548,848,871 bp, with a weighted mean read length of 412.27 bp and 40,522–158,872 reads per sample. Alpha diversity was estimated from OTUs defined at 97% sequence similarity (Figure 2). ACE and Chao 1 describe community richness, whereas Shannon and Simpson capture diversity and evenness. Good’s coverage approached 1.0 in every group, supporting adequate detection of the dominant sequence diversity within samples. Day-0 samples had the largest observed ACE, Chao 1, Shannon, and Sobs values, while these indices generally declined during storage and Simpson moved in the opposite direction. These patterns are compatible with increasing dominance by fewer taxa. Before downstream analysis, all samples were normalized by equal-depth subsampling to 40,137 sequences per sample, thereby substantially reducing the direct influence of differences in raw sequencing depth. Nevertheless, stochastic subsampling and OTU filtering may still affect the detection of low-abundance OTUs. At matched storage times, CK had lower ACE, Chao 1, Shannon, and Sobs values than PS-NPs, indicating differences in the reported alpha-diversity metrics [31,32].

#### 3.1.2. Beta Diversity of Bacterial Communities

**Figure 3:**
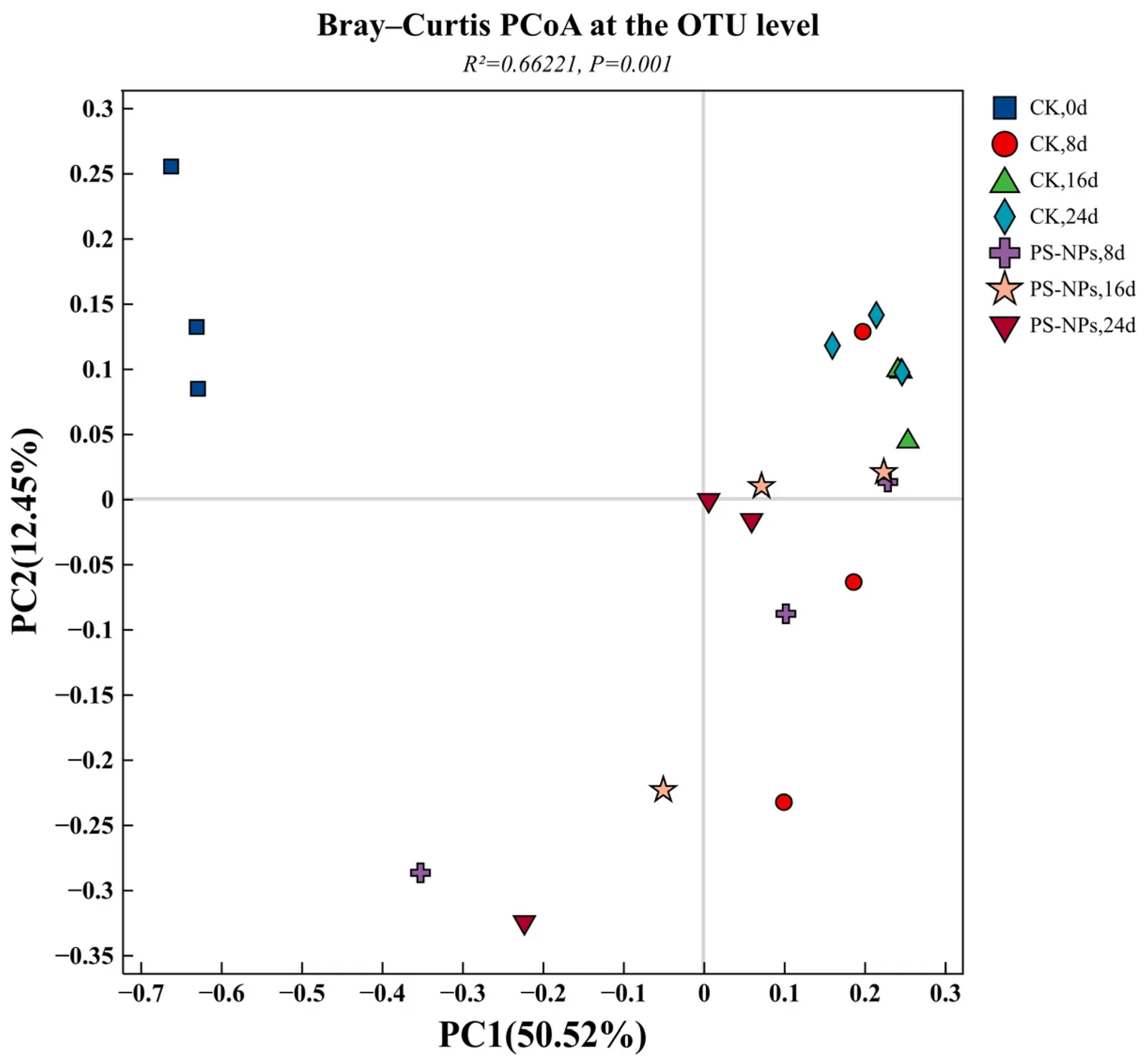
Principal-coordinate analysis of bacterial communities based on Bray–Curtis dissimilarities at the OTU level.

Principal-coordinate analysis (PCoA) based on Bray–Curtis dissimilarities revealed structured variation in bacterial community composition among the seven observed treatment–time groups at the OTU level (Figure 3). PC1 and PC2 explained 50.52% and 12.45% of the total variation, respectively, accounting for 62.97% collectively. The day-0 CK samples formed a compact cluster separated from the stored samples primarily along PC1, whereas partial overlap occurred among the stored CK and PS-NPs groups.

PERMANOVA showed an overall difference in bacterial community composition among the seven groups (pseudo-F(6, 14) = 4.574, R^2^ = 0.662, *p* = 0.001). The seven-group classification accounted for 66.2% of the variation in Bray–Curtis dissimilarities. PERMDISP detected no significant heterogeneity in multivariate dispersion (F(6, 14) = 0.711, *p* = 0.4985), indicating that the PERMANOVA result was unlikely to be driven by unequal within-group dispersion. Collectively, these beta-diversity results demonstrate overall restructuring of the bacterial community across the observed treatment–time conditions. However, the omnibus PERMANOVA does not identify which specific group pairs differed significantly or independently quantify the effects of treatment and storage time.

#### 3.1.3. Relationship with Alpha Diversity and Taxonomic Composition

The beta-diversity results complemented the alpha-diversity analysis by describing differences in community composition among samples rather than richness and evenness within individual samples. The separation of the day-0 CK samples from the stored samples was consistent with substantial restructuring of the bacterial community during storage. This pattern complemented the storage-related changes observed in the Ace, Chao, Shannon, Simpson, and Sobs indices. However, changes in alpha-diversity metrics should not be interpreted as direct evidence of ecological loss, because alpha diversity and beta diversity quantify different aspects of community structure.

At matched storage times, the partial overlap between CK and PS-NPs samples in the PCoA indicated that differences in richness and diversity did not necessarily correspond to complete separation in overall community composition. Therefore, the PCoA and PERMANOVA results support an overall restructuring of the bacterial community across the observed treatment–time groups, while the phylum- and genus-level abundance profiles are required to identify the taxa associated with these differences.

#### 3.1.4. Differential Analysis of Bacterial Communities

**Figure 4:**
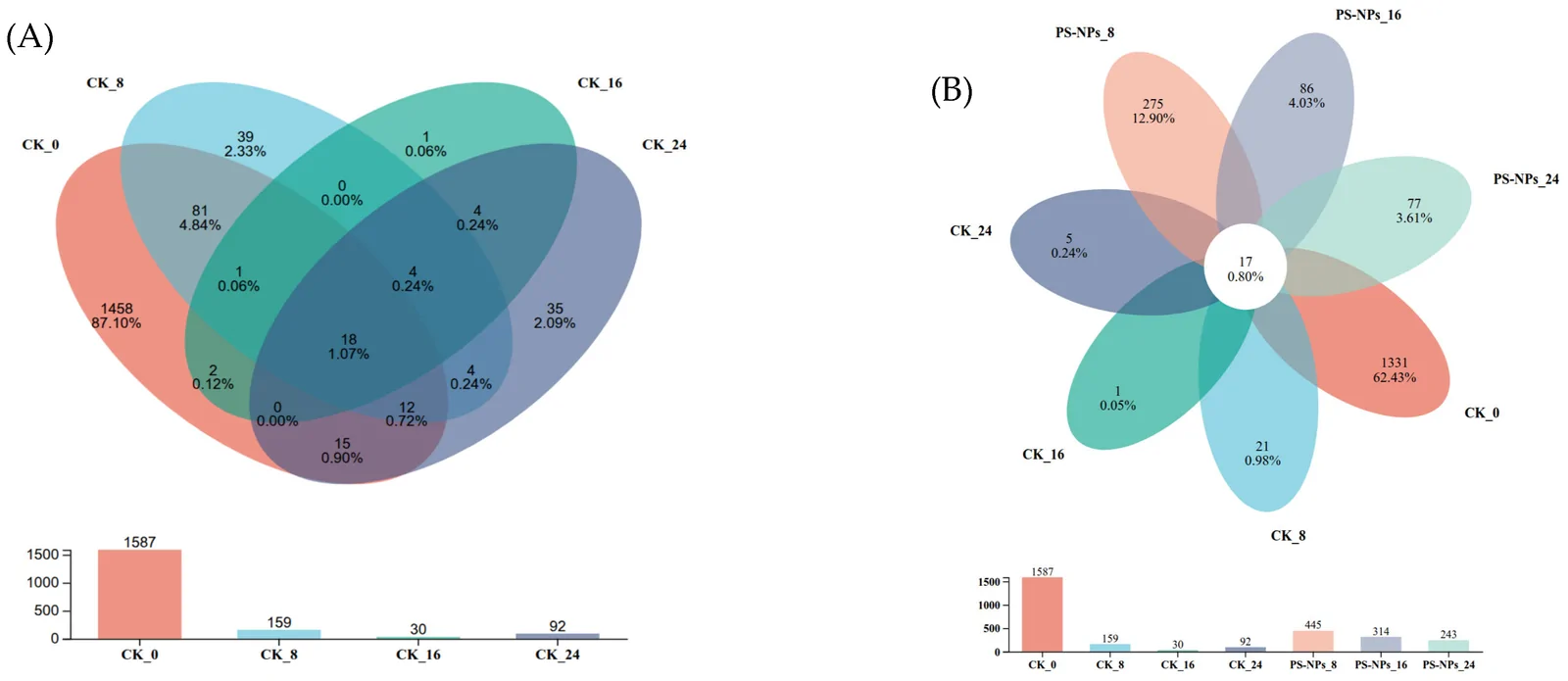
Distribution of operational taxonomic units (OTUs) during storage: (**A**) total, unique, and shared OTUs in CK samples at days 0, 8, 16, and 24; (**B**) total, unique, and shared OTUs across all CK and PS-NPs treatment-time groups.(The bar chart shows the total number of OTUs detected at 0, 8, 16, and 24 days of storage, whereas the Venn diagram shows the numbers of unique and shared OTUs across these time points. Of the 1587 OTUs detected on day 0, 1458 were unique to this time point and 129 were shared with at least one later time point).

Venn diagrams based on OTU clustering summarized group-level OTU overlap across storage times and treatments (Figure 4A,B). In CK, 1587, 159, 30, and 92 OTUs were detected on days 0, 8, 16, and 24, respectively. These values represent presence–absence unions across the three biological replicates at each time point rather than mean OTU counts per sample. Of the 1587 OTUs detected at CK day 0, 1458 were unique to this group, whereas the remaining 129 occurred in at least one later CK group (Figure 4A). Across the four CK time points, 18 OTUs were shared, while 1458, 39, 1, and 35 OTUs were unique to days 0, 8, 16, and 24, respectively. Thus, the values of 1587 and 1458 represent the total and day-0-specific OTU counts, respectively, and are fully consistent with Figure 4A. For PS-NPs, 445, 314, and 243 OTUs were detected on days 8, 16, and 24, respectively. Figure 4B further shows that 17 OTUs were shared across all seven observed treatment–time groups. These Venn diagrams were used only to describe OTU overlap and were not used for statistical inference.

Although filtered-read counts varied among the original libraries, all OTU-abundance tables were rarefied to 40,137 reads per sample before the Venn analysis. The original read depth ranged from 76,682 to 158,872 reads for CK day 0 and from 56,917 to 69,840 reads for CK day 8. Equal-depth subsampling substantially reduced the direct influence of unequal sequencing depth on OTU detection. However, the original random seed was not archived, and stochastic subsampling and OTU filtering may have affected the detection of low-abundance OTUs. The group-level OTU count decreased by approximately 90.0% from CK day 0 to day 8. This decrease represents a reduction in observed OTU richness rather than an equivalent decline in bacterial biomass or the confirmed disappearance of 1428 bacterial taxa. The same trend was evident at the individual-sample level, with Sobs decreasing from 538–1095 at day 0 to 86–106 at day 8. This decline was accompanied by lower Shannon diversity and greater Simpson dominance, whereas culturable bacterial counts increased by approximately 1 log_10_ CFU/g. Collectively, these findings are consistent with increasing dominance by a limited subset of taxa and reduced detection of less abundant community members during storage. Accordingly, the Venn counts provide descriptive evidence of community restructuring, rather than direct evidence of taxon extinction or reduced total bacterial abundance. Overall compositional differences were evaluated independently using Bray–Curtis PCoA and PERMANOVA (Figure 3).

#### 3.1.5. Bacterial Community Composition Analysis

**Figure 5:**
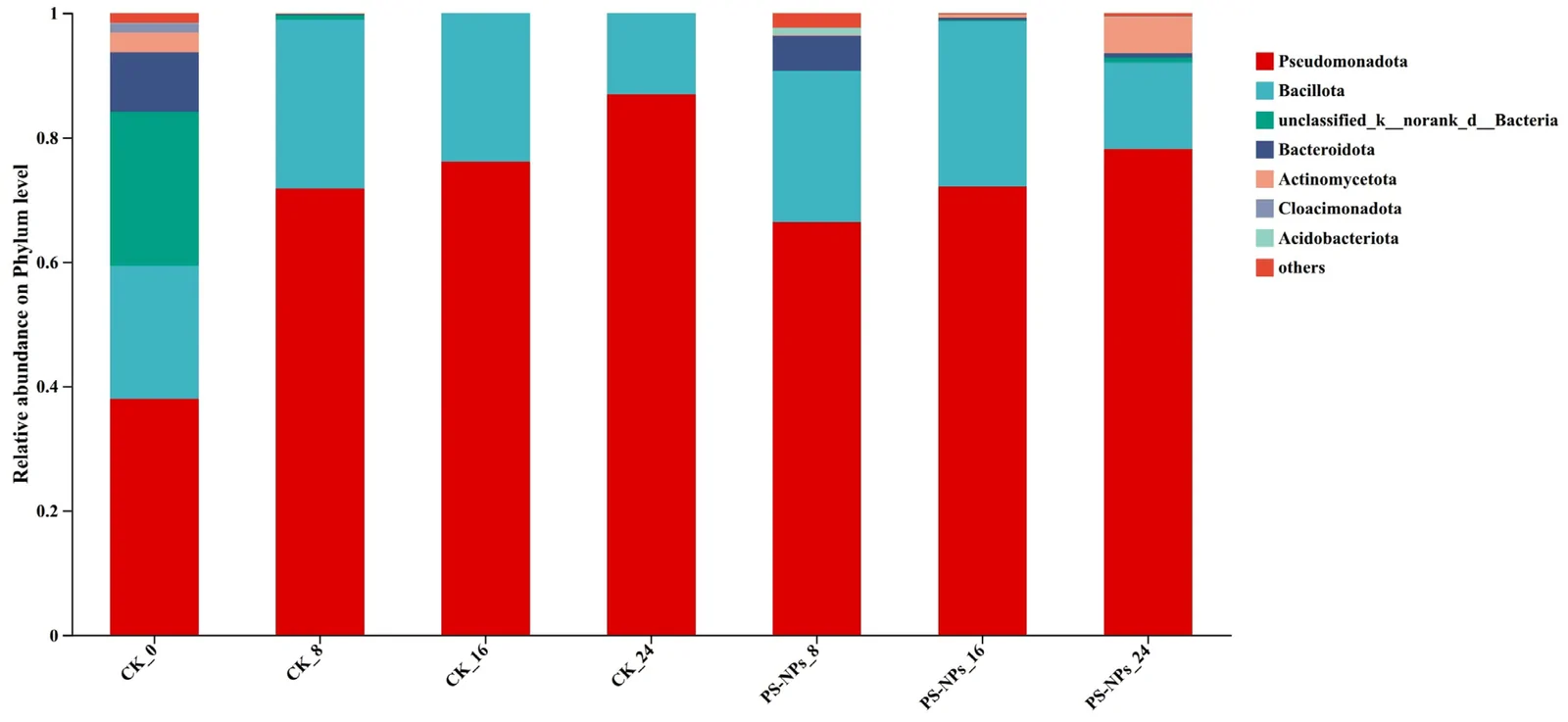
Relative abundance at the phylum level under different storage conditions.

Figure 5 summarizes phylum-level relative abundance across storage times and pad treatments. Seven bacterial phyla were detected. Pseudomonadota, Bacillota, and Bacteroidota together represented 68.87% of the initial community, contributing 37.97%, 21.37%, and 9.53%, respectively. Unclassified bacteria accounted for another 24.75%, potentially reflecting environmental inputs and unresolved taxonomy [33]. In CK, Pseudomonadota and Bacillota increased with storage and together exceeded 98% relative abundance by day 8. The convergence toward Pseudomonadota and Bacillota is biologically plausible because cold-tolerant members of these phyla can remain metabolically active in oxygenated meat packages and exploit soluble nutrients released into surface exudate. Thus, the phylum-level pattern indicates selective enrichment under the storage environment rather than uniform proliferation of the initial community.

**Figure 6:**
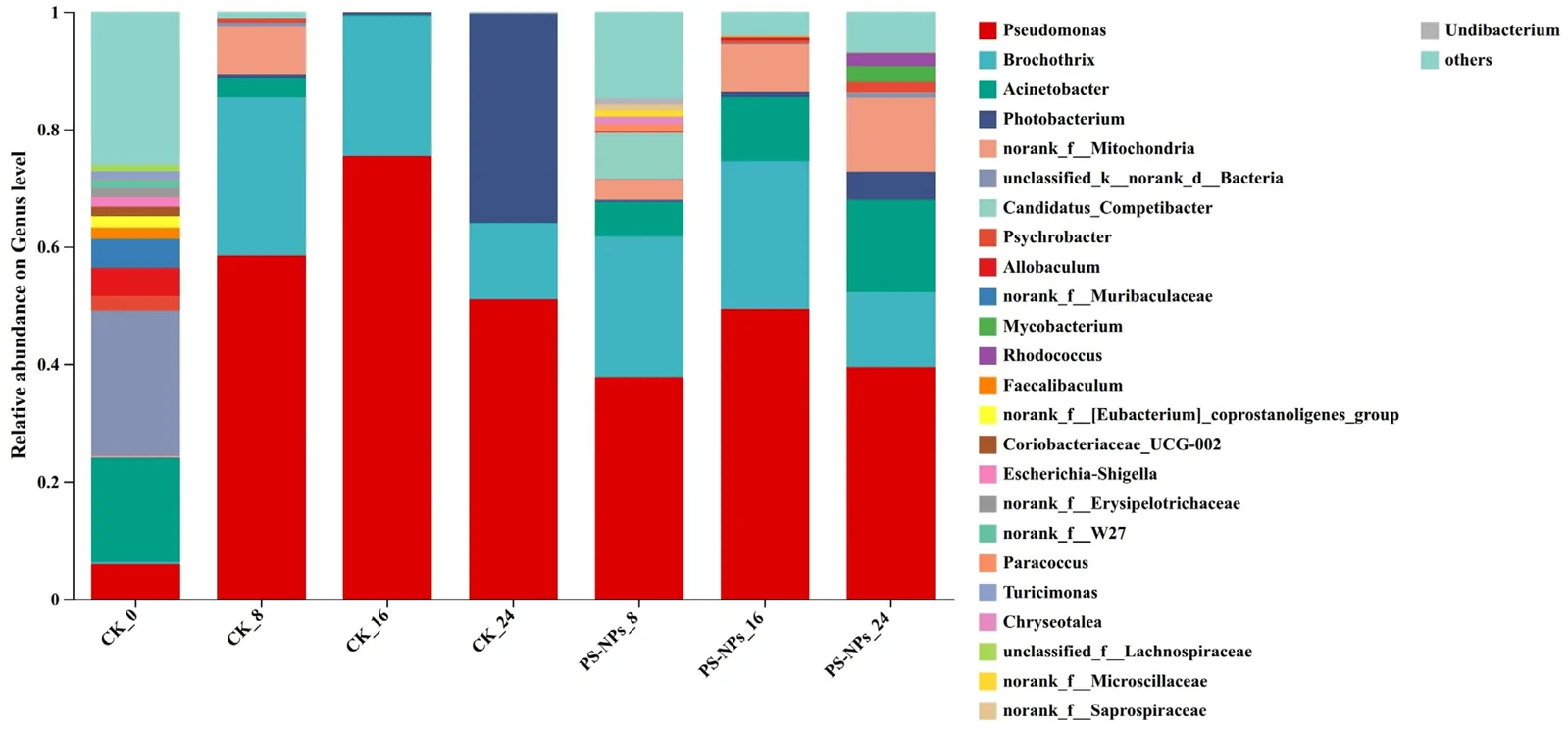
Relative abundance at the genus level under different storage conditions.

Pseudomonadota progressively dominated CK, while Bacillota changed less and declined slightly by day 24. Pseudomonadota also increased under PS-NPs, but its relative abundance remained below CK and reached 78.16% on day 24. This pattern describes a treatment-associated difference in relative community profiles [34]. However, 16S rRNA gene sequencing produces compositional data, so a lower relative abundance cannot by itself establish lower absolute cell numbers or direct growth inhibition [35]. Figure 6 presents the corresponding genus-level profiles. Initially, *Pseudomonas*, *Acinetobacter*, unclassified bacteria, and norank_f__Muribaculaceae accounted for 5.93%, 17.69%, 24.75%, and 4.87%, respectively. Storage coincided with increasing dominance by a smaller number of genera [36]. By day 8, *Pseudomonas* and *Brochothrix* had become dominant in CK, reaching 58.46% and 26.99%, respectively. The smaller Pseudomonadota fraction under PS-NPs could arise if sustained OEO exposure altered membrane function and relative growth rates while the absorbent matrix reduced the water-rich surface niche. Because those effects would act simultaneously on several taxa and on the package microenvironment, the observed difference is better interpreted as community restructuring than as direct killing of one phylum.

**Figure 7:**
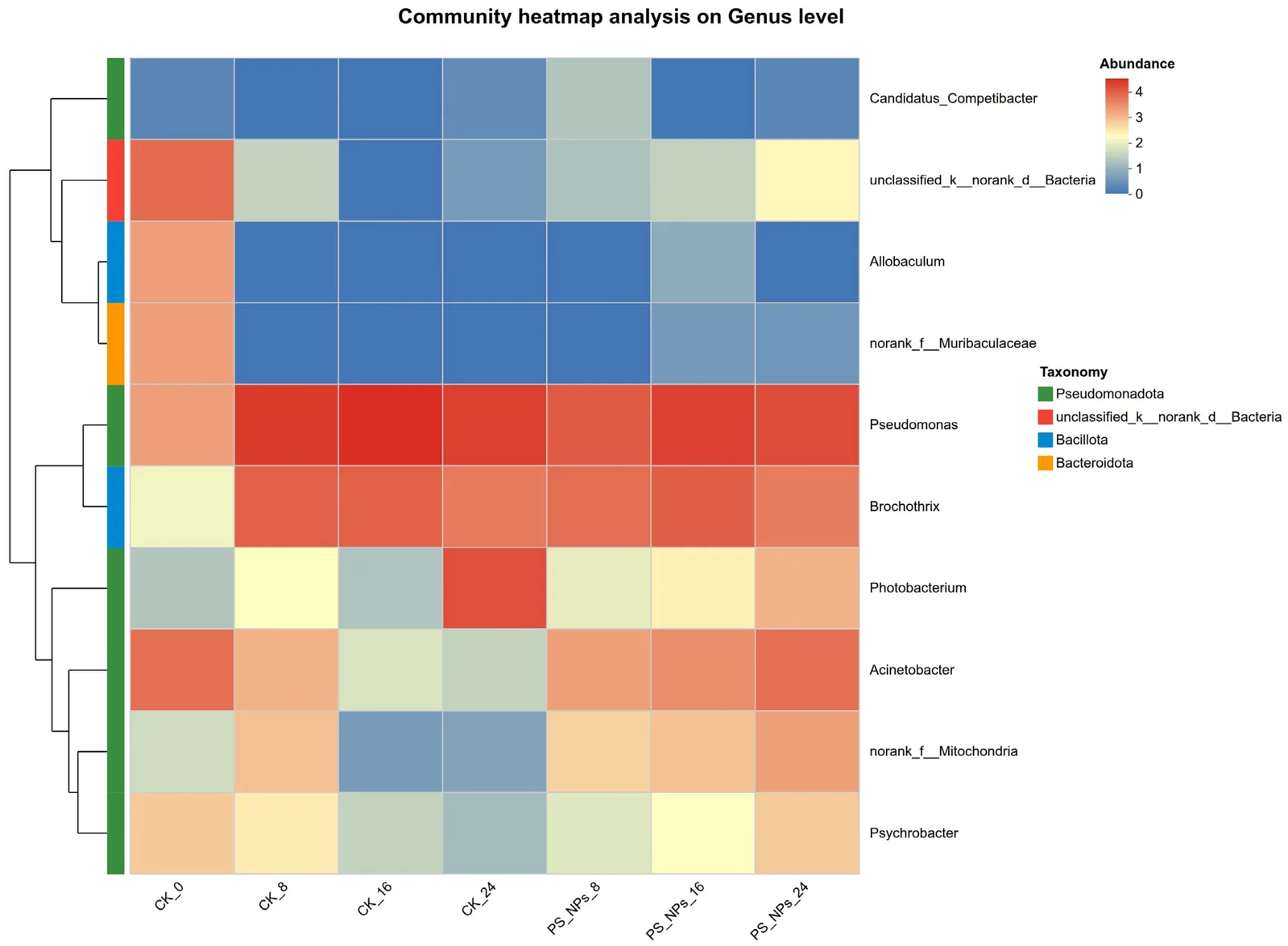
Heatmap of genus-level bacterial profiles across storage treatments.

In CK, *Pseudomonas* reached 75.38% on day 16, while *Brochothrix* was 23.87%. By day 24, *Pseudomonas* had decreased to 51.02% but remained the leading genus while *Photobacterium* increased to 35.57%. Under PS-NPs, *Pseudomonas* and *Brochothrix* accounted for 37.77% and 23.97% on day 8. Their highest relative abundances occurred on day 16 at 49.34% and 25.18%, followed by *Acinetobacter* at 10.92%. On day 24, *Pseudomonas* and *Brochothrix* were 39.48% and 12.70%, whereas *Acinetobacter* was 15.75%. Thus, *Pseudomonas* followed a similar temporal pattern in both treatments but represented a smaller fraction of the PS-NPs community. This result reflects relative community composition only. Figure 7 compares the ten most abundant taxa by heatmap and hierarchical clustering.

No single genus dominated the day-0 pork community. As storage progressed, *Pseudomonas*, *Brochothrix*, and *Acinetobacter* became the principal genera, although their proportions varied among treatments. *Pseudomonas* and *Acinetobacter* are Gram-negative and possess an outer membrane that limits the access of nisin to lipid II in the cytoplasmic membrane [37]. *Brochothrix* is Gram-positive and lacks this outer-membrane barrier, allowing more direct nisin-lipid II interactions. OEO phenolics can permeabilize bacterial membranes [16], which could complement nisin activity after barrier disruption. Nevertheless, envelope composition, efflux, biofilm formation, cold adaptation, and competition among taxa can all influence community proportions. The lower *Pseudomonas* proportion therefore does not establish greater intrinsic susceptibility than *Acinetobacter* or *Brochothrix*. Isolate-level MIC or kill-curve testing and absolute taxon quantification would be required to support that conclusion.

### 3.2. Dynamic Changes of Physicochemical Quality and Oxidative Stability in Pork

#### 3.2.1. Changes in TVB-N of Fresh Pork Under Different Storage Conditions

**Figure 8:**
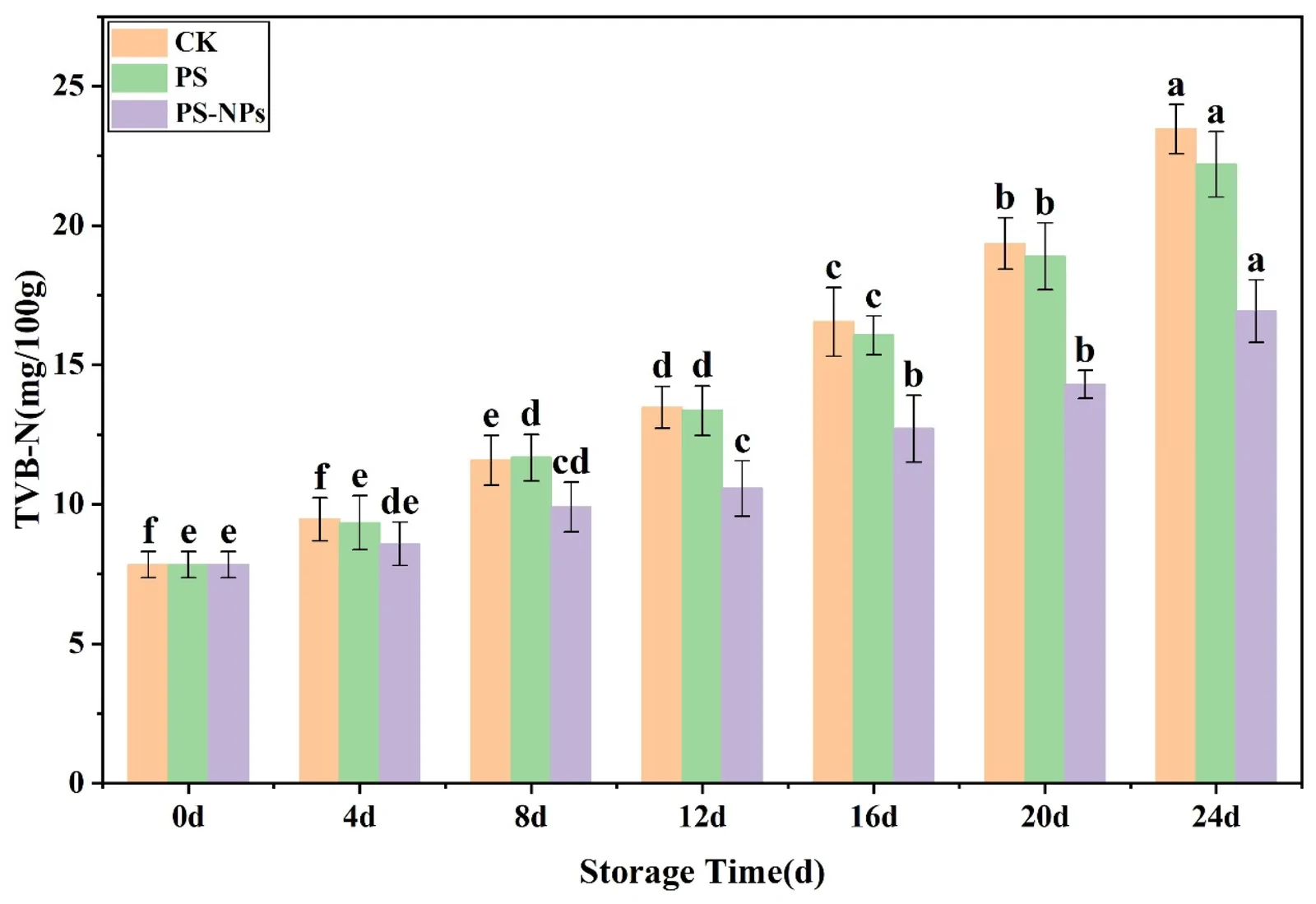
Changes in total volatile basic nitrogen of fresh pork under different storage conditions. Data are presented as mean ± standard deviation (SD; *n* = 3). Different lowercase letters above the bars indicate significant differences among treatment–time groups (*p* < 0.05).

TVB-N rose in every group but was lower under PS-NPs than under CK or PS (*p* < 0.05; Figure 8). On day 16, CK and PS reached 16.54 and 16.06 mg/100 g, respectively, exceeding the acceptance limit. By day 24, PS reached 22.19 mg/100 g, compared with 23.457 mg/100 g in CK. Absorption of pork exudate by the PS pad may have made conditions less favorable for spoilage organisms and slowed TVB-N formation [38]. Nevertheless, PS alone did not prevent threshold exceedance by day 16. In the PS-NPs group, TVB-N was 14.29 mg/100 g on day 20 and crossed the Chinese-standard limit only at day 24. The slower TVB-N accumulation under PS-NPs coincided with lower Pseudomonas relative abundance on day 24 (39.48% versus 51.02% in CK). This concordance supports an association between bacterial proliferation and nitrogenous spoilage. Mechanistically, TVB-N accumulates when microbial and endogenous proteolysis releases amino acids that are subsequently deaminated to ammonia and other volatile bases. The faster increase in CK is therefore consistent with its higher cultivable count and stronger dominance by spoilage-associated taxa, whereas the delayed increase under PS-NPs is consistent with slower substrate turnover. Untreated superchilled pork has likewise shown parallel increases in aerobic counts and TVB-N, with lower storage temperatures delaying both responses [17]. This agreement is consistent with a possible microbial contribution to the observed TVB-N trajectory, although this interpretation remains tentative.

#### 3.2.2. Changes in TVC of Fresh Pork Under Different Storage Conditions

**Figure 9:**
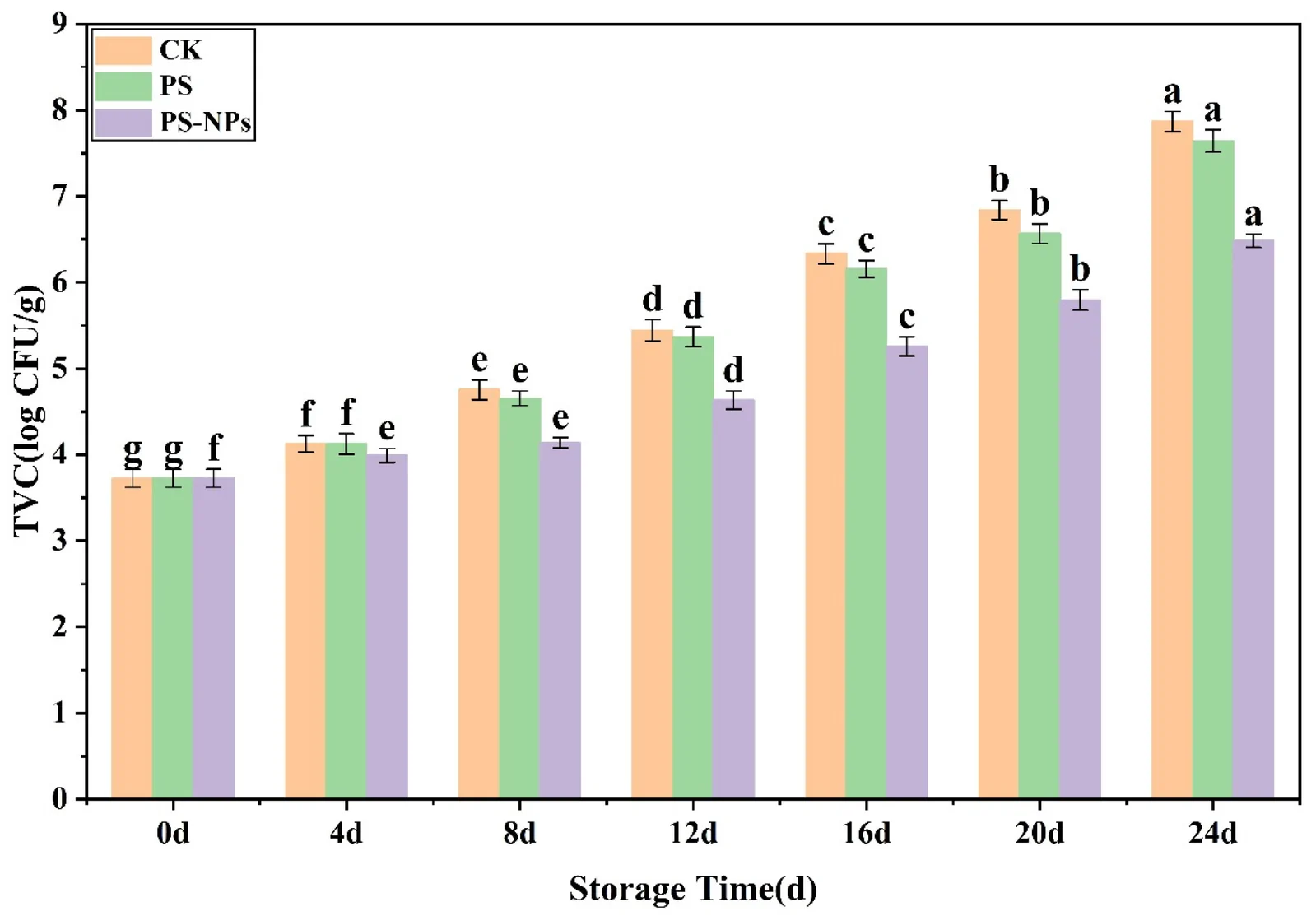
Changes in total viable count of fresh pork under different storage conditions. Data are presented as mean ± standard deviation (SD; *n* = 3). Different lowercase letters above the bars indicate significant differences among treatment–time groups (*p* < 0.05).

TVC increased with storage in all treatments and followed a pattern similar to TVB-N (Figure 9). CK and PS exceeded 6 log CFU/g on day 16, with counts of 6.33 and 6.16 log CFU/g, respectively. PS remained below CK during later storage, possibly because exudate absorption reduced surface moisture available for microbial growth. PS-NPs did not reach 6.48 log CFU/g until day 24, indicating slower accumulation of cultivable bacteria. Carvacrol- and thymol-rich OEO can partition into bacterial membranes, disrupt permeability, and impair proton and ion homeostasis [16]. Nisin binds lipid II, inhibits peptidoglycan synthesis, and forms membrane pores in susceptible Gram-positive cells [39]. Gram-negative outer membranes restrict nisin access, although membrane permeabilization and nanoscale delivery can broaden its activity [36]. Within ONSNPs and the PVA/SPI matrix, encapsulation may protect volatile OEO, improve dispersion, and sustain release at the food-package interface [14,15]. These complementary mechanisms are plausible, but the present study did not compare free and encapsulated antimicrobials or perform taxon-specific kill assays. At −1 °C, sufficient unfrozen water remains available for psychrotrophic cells, so temperature control delays rather than eliminates proliferation. The separation between PS-NPs and the nanoparticle-free PS group suggests that moisture absorption alone was insufficient and that sustained antimicrobial delivery contributed additional pressure. This interpretation agrees with control studies in which lower or more stable superchilling temperatures slowed aerobic-count increases, but realistic cold-chain conditions still permitted time-dependent proliferation [17,20].

#### 3.2.3. Changes in TBARS of Fresh Pork Under Different Storage Conditions

**Figure 10:**
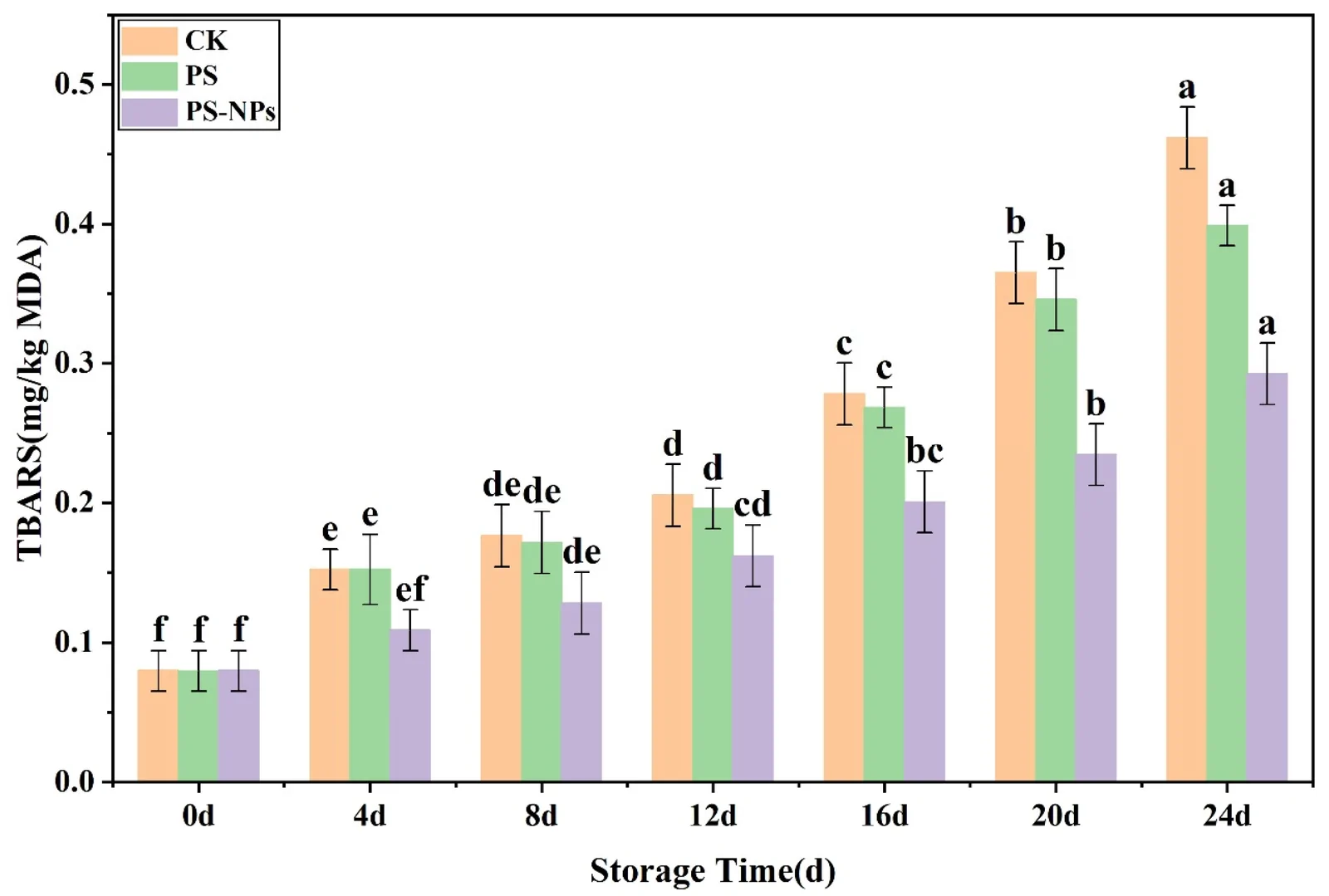
Storage-dependent TBARS values of pork subjected to CK, PS, and PS-NPs treatments. Data are presented as mean ± standard deviation (SD; *n* = 3). Different lowercase letters above the bars indicate significant differences among treatment–time groups (*p* < 0.05).

TBARS increased in all groups during storage (Figure 10). In CK, TBARS increased from 0.080 to 0.46 mg MDA/kg by day 24, representing a 475% increase. The corresponding day-24 values were 0.40 mg MDA/kg in PS and 0.29 mg MDA/kg in PS-NPs. Thus, the PS-NPs value was 36.96% lower than CK and 27.5% lower than PS. These results indicate that PS-NPs delayed lipid oxidation [40]. The limited difference between CK and PS indicates that exudate absorption alone provided only modest protection against lipid peroxidation. In contrast, the larger reduction under PS-NPs is consistent with carvacrol- and thymol-mediated interruption of radical propagation and with slower release of pro-oxidative metabolites as microbial growth was delayed. Recent untreated-pork studies show that deeper and more stable superchilling slows TBARS formation, whereas temperature fluctuations enhance oxidation and free-water migration [17,18,19]. The present treatment effect therefore operates in the same deterioration pathway but adds antioxidant protection beyond temperature control; direct radical-scavenging and metal-chelation measurements would be required to verify the proposed route.

#### 3.2.4. Changes in Carbonyl Content of Fresh Pork Under Different Storage Conditions

**Figure 11:**
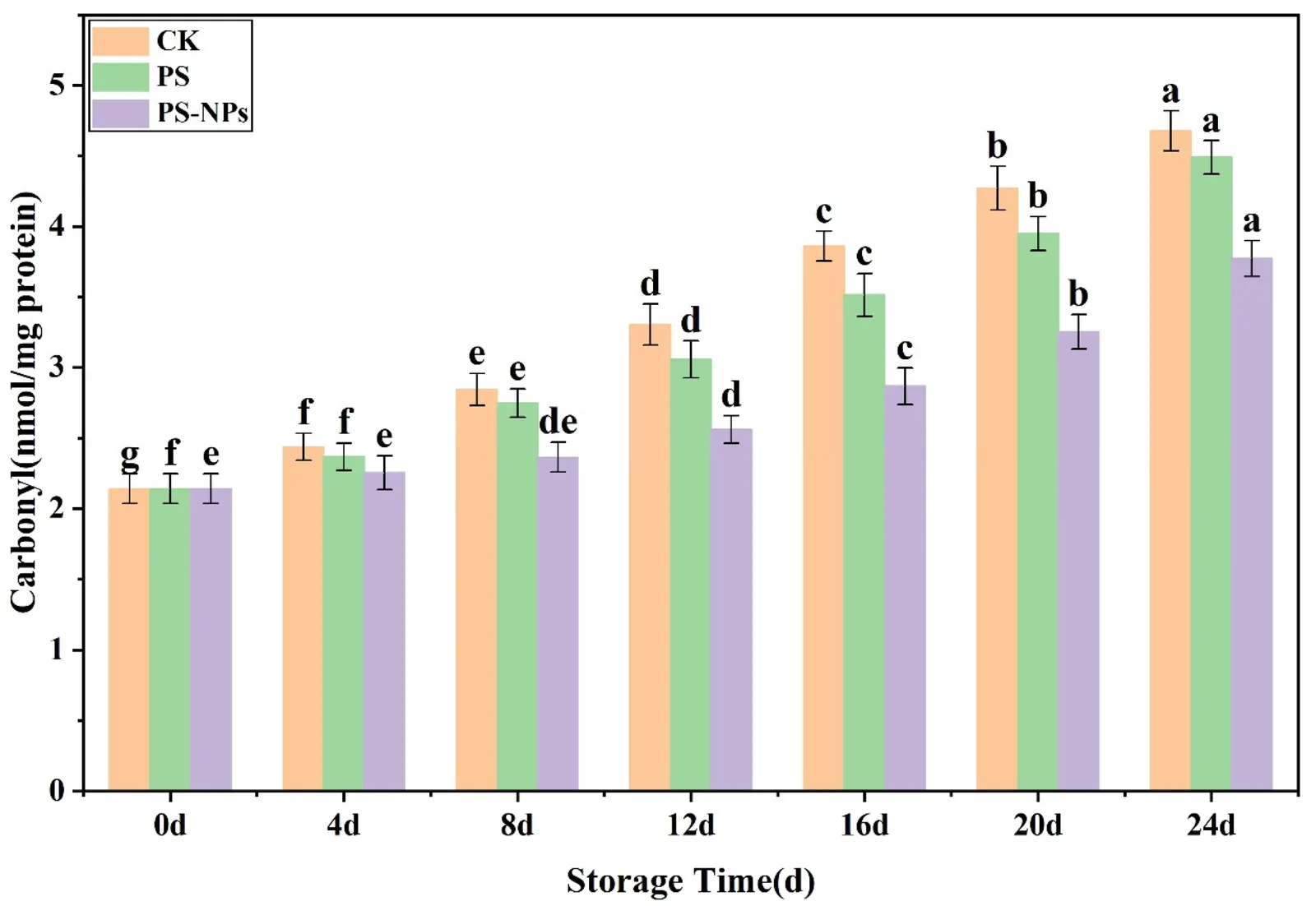
Changes in carbonyl content of fresh pork under different storage conditions. Data are presented as mean ± standard deviation (SD; *n* = 3). Different lowercase letters above the bars indicate significant differences among treatment–time groups (*p* < 0.05).

Protein carbonyls increased during storage in all treatments (Figure 11). CK reached 4.68 nmol/mg protein on day 24, a 118.69% increase from the initial 2.14 nmol/mg protein. The final values were 4.49 nmol/mg protein for PS and 3.77 nmol/mg protein for PS-NPs. Protein and lipid oxidation are interconnected in muscle foods. Lipid-derived peroxyl and alkoxyl radicals can attack amino-acid side chains, while secondary aldehydes can form protein carbonyls and cross-links [35,41,42,43]. One mechanistic hypothesis is that the lower TBARS value under PS-NPs reflected a smaller lipid-oxidation burden and therefore fewer lipid-derived oxidants available for protein carbonyl formation. A second hypothesis is that OEO-mediated radical scavenging or metal chelation affected both oxidative pathways [44]. Because radicals, reactive aldehydes, and metal-dependent redox processes were not measured directly, the present study cannot establish either proposed pathway. The lower carbonyl content in PS-NPs was directionally consistent with reports of slower protein oxidation under more effective superchilling conditions [17]. However, this comparison does not identify the underlying mechanism. The concurrent reductions in TBARS and carbonyls are compatible with possible lipid-to-protein oxidative coupling, but their parallel behavior does not demonstrate a causal relationship. The nanoparticle-free PS treatment produced only a modest carbonyl reduction, suggesting that absorbency alone may not fully account for the response observed with PS-NPs. Nevertheless, contributions from other unmeasured physicochemical factors cannot be excluded.

### 3.3. Effects of Active Pads on the Physical Quality and Sensory Characteristics of Pork

#### 3.3.1. Change in Color of Fresh Pork Under Different Storage Conditions

Table 1 reports L*, a*, and b* across treatments and storage times. Lightness generally declined during storage. CK decreased from 54.50 to 50.16, while PS remained lower than CK during most of the storage period and ended at 48.63. Exudate uptake by the PS pad may have reduced surface reflectance [45]. PS-NPs showed modest L* fluctuations and finished at 52.23, above CK and PS, indicating better retention of surface lightness. Redness first increased and then declined in all treatments. CK rose from 6.74 to 9.78 on day 8 before falling to 4.26, whereas PS peaked at 9.05 and ended at 4.88. PS-NPs finished at an a* value of 8.08. The final b* values were 16.55, 16.20, and 14.90 for CK, PS, and PS-NPs, respectively. These observations show better retention of measured color coordinates under PS-NPs, but they do not identify the underlying mechanism. Reduced oxygen consumption and slower metmyoglobin formation are hypotheses that could explain the a* pattern. Neither oxygen consumption nor myoglobin redox state was measured in this study. The lower b* value likewise cannot by itself establish reduced lipid oxidation.

#### 3.3.2. Changes in Drip Loss of Fresh Pork Under Different Storage Conditions

**Figure 12:**
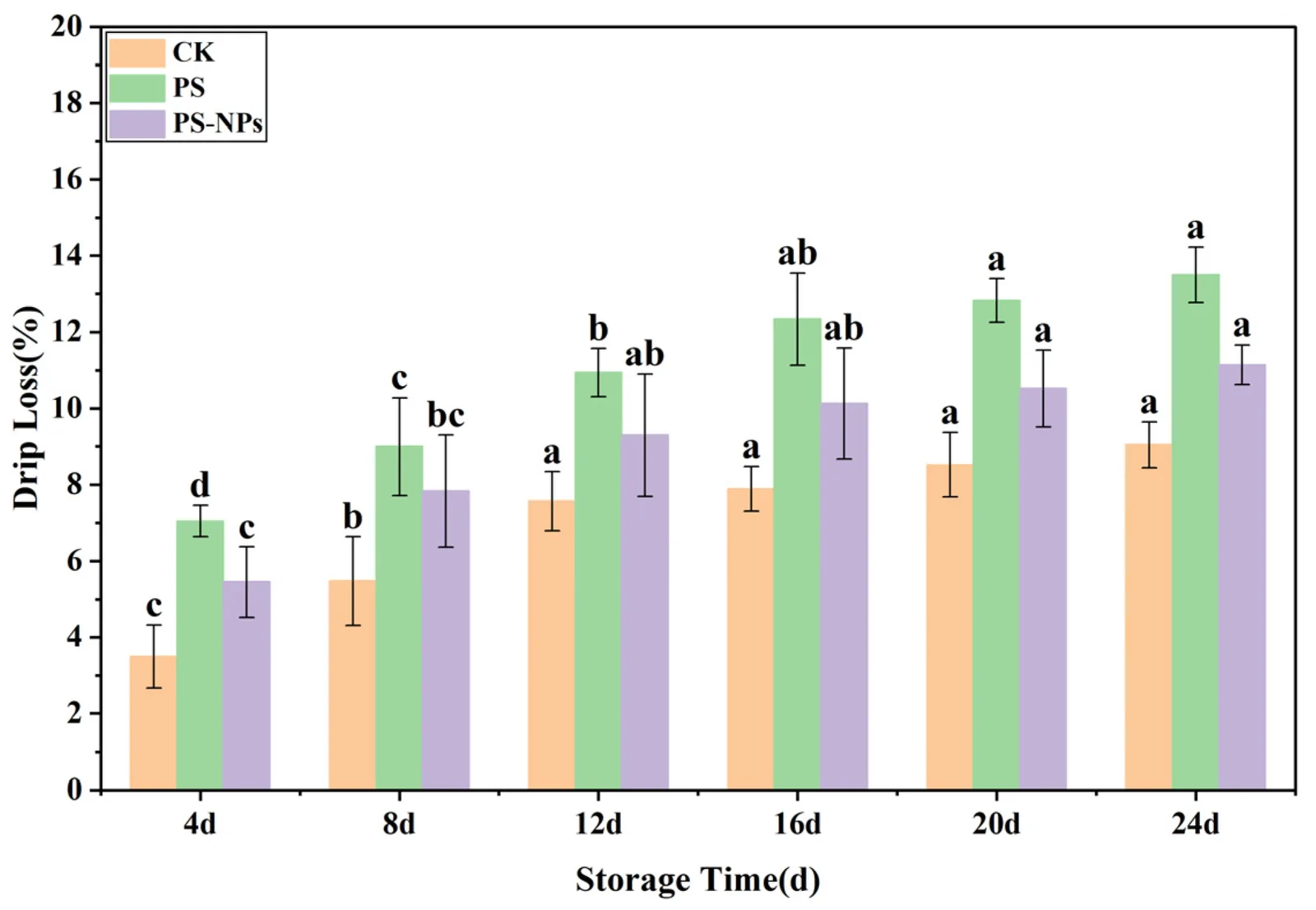
Changes in drip loss of fresh pork under different storage conditions. Data are presented as mean ± standard deviation (SD; *n* = 3). Different lowercase letters above the bars indicate significant differences among treatment–time groups (*p* < 0.05).

Fresh pork has high water activity, so moisture migration and exudate accumulation are important determinants of storage quality. Drip loss increased with storage in all treatments (*p* < 0.05; Figure 12). On day 4, the drip loss was 3.50%, 7.05%, and 5.45% in CK, PS, and PS-NPs, respectively. PS remained higher than the other groups and reached 13.50% at the end of storage, possibly because the absorbent pad rapidly removed liquid from the pork surface. The drip loss under PS-NPs remained below PS, which may reflect a balance between pad absorption and slower deterioration of the tissue water-holding matrix. This pattern coincided with slower increases in TVC and protein carbonyl content under PS-NPs. However, microbial protease activity, myofibrillar degradation, and absolute taxon counts were not measured. The drip-loss result therefore cannot be attributed specifically to inhibition of *Brochothrix* or *Pseudomonas*. This interpretation is supported by untreated superchilling studies in which temperature fluctuations produced repeated ice melting and recrystallization, widened structural channels, shifted immobilized water toward freer states, and increased drip loss [18,19]. Although storage at −1 °C is less severe than those fluctuation regimes, the same structure–water relationship explains why oxidative or proteolytic weakening could amplify exudation during prolonged storage.

#### 3.3.3. Changes in Cooking Loss of Fresh Pork Under Different Storage Conditions

**Figure 13:**
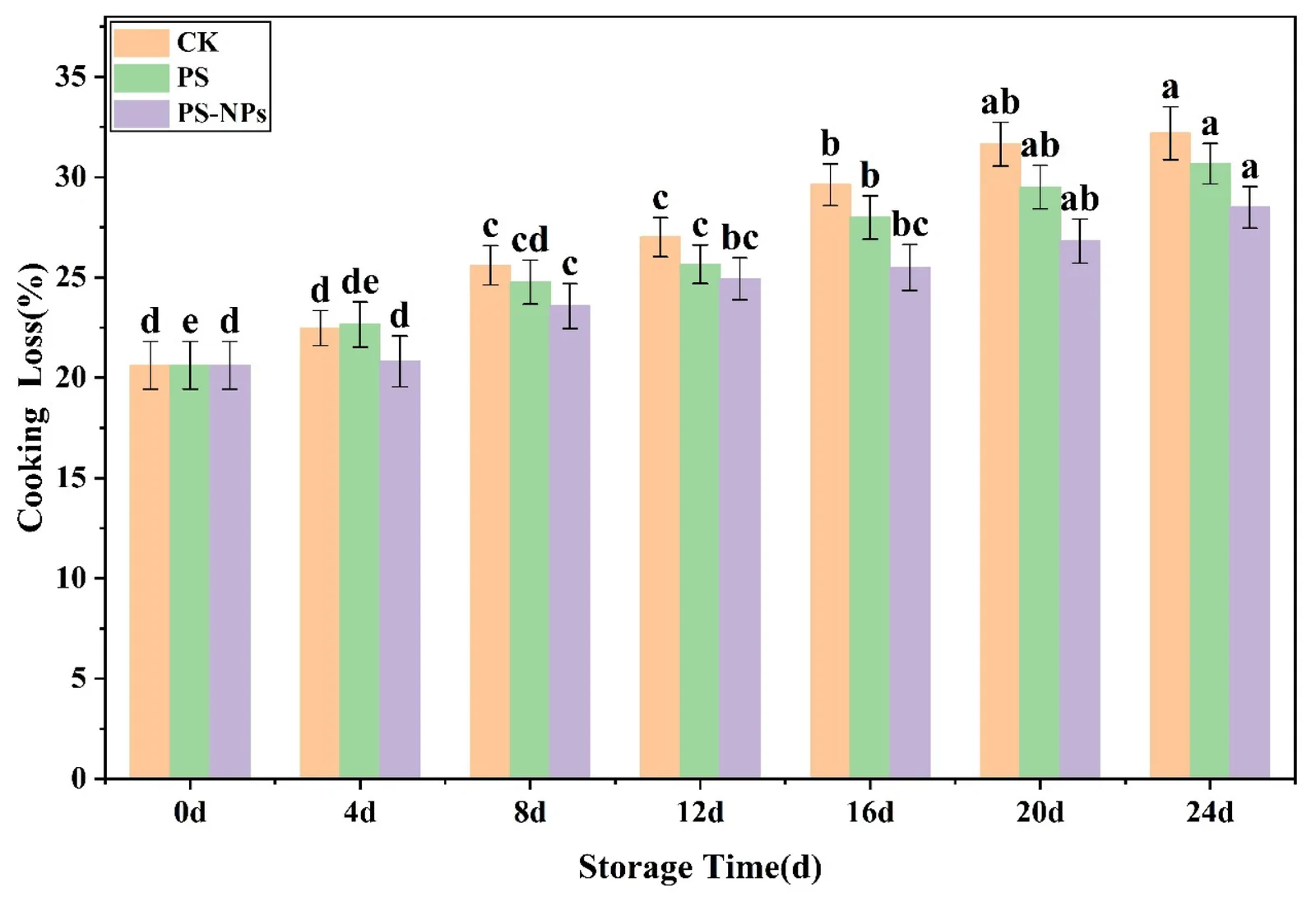
Changes in cooking loss of fresh pork under different storage conditions. Data are presented as mean ± standard deviation (SD; *n* = 3). Different lowercase letters above the bars indicate significant differences among treatment–time groups (*p* < 0.05).

Cooking loss increased throughout storage, indicating progressive impairment of pork water-holding capacity (Figure 13). CK increased from 20.62% to 32.18%, while PS and PS-NPs reached 30.66% and 28.49% on day 24, respectively. A working mechanistic hypothesis is that microbial or endogenous proteolysis weakened myofibrillar and cytoskeletal proteins while protein oxidation promoted carbonyl formation, cross-linking, aggregation, and reduced protein solubility [41,42]. Such changes could alter myofibrillar spacing and protein-water interactions, allowing heat-induced contraction to expel more water. The lower TVC and carbonyl content under PS-NPs were consistent with better retention of myofibrillar functionality and lower cooking loss. However, protease activity, protein conformation, and myofibrillar microstructure were not measured, so this explanation is a hypothesis rather than a mechanism demonstrated in the present study.

#### 3.3.4. Changes in Sensory Properties of Fresh Pork Under Different Storage Conditions

**Figure 14:**
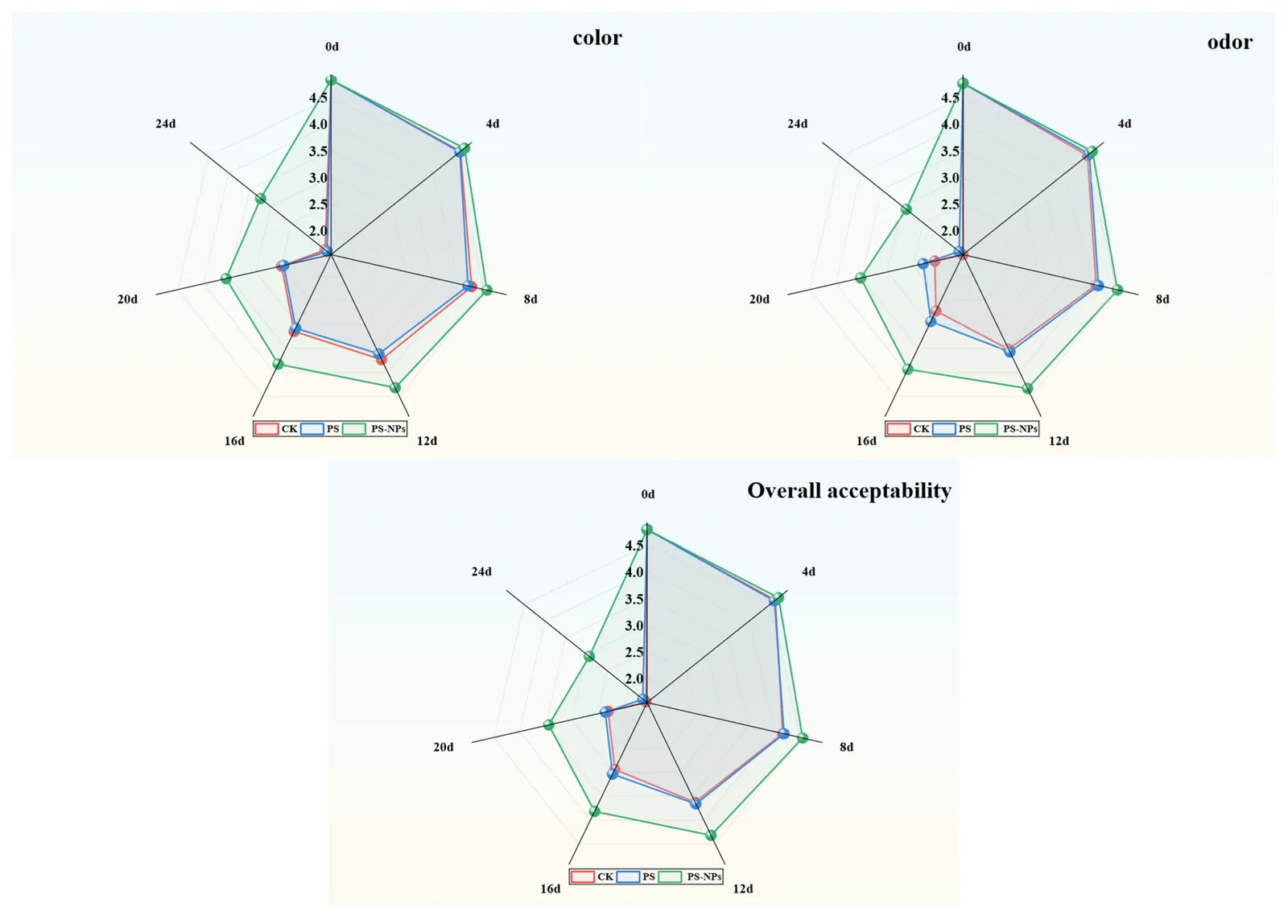
Changes in sensory evaluation of fresh pork under different storage conditions.

Sensory scores decreased progressively in all treatments (Figure 14). By day 16, odor and overall acceptability in CK were below 3, while the color score approached the acceptance threshold. The PS odor score also became unacceptable on day 16, and its other scores approached 3. After 24 days, CK and PS scores were below 2, and panelists perceived pronounced undesirable odors. Because volatile compounds were not measured, these odors cannot be assigned specifically to protein degradation, lipid oxidation, or individual microbial metabolites. In PS-NPs, odor and overall acceptability did not fall below 3 before day 24, while color remained close to the threshold. The sensory results were therefore consistent with slower overall deterioration under PS-NPs, without identifying the chemical origin of odor changes. The delayed sensory decline tracked the lower TVC, TVB-N, TBARS, and carbonyl values, indicating that panelists perceived the integrated consequence of slower microbial and oxidative deterioration.

### 3.4. Exploratory Correlation Analysis and Hypothesis Generation

#### 3.4.1. Associations Between Dominant Taxa and Chemical Quality Indices

**Figure 15:**
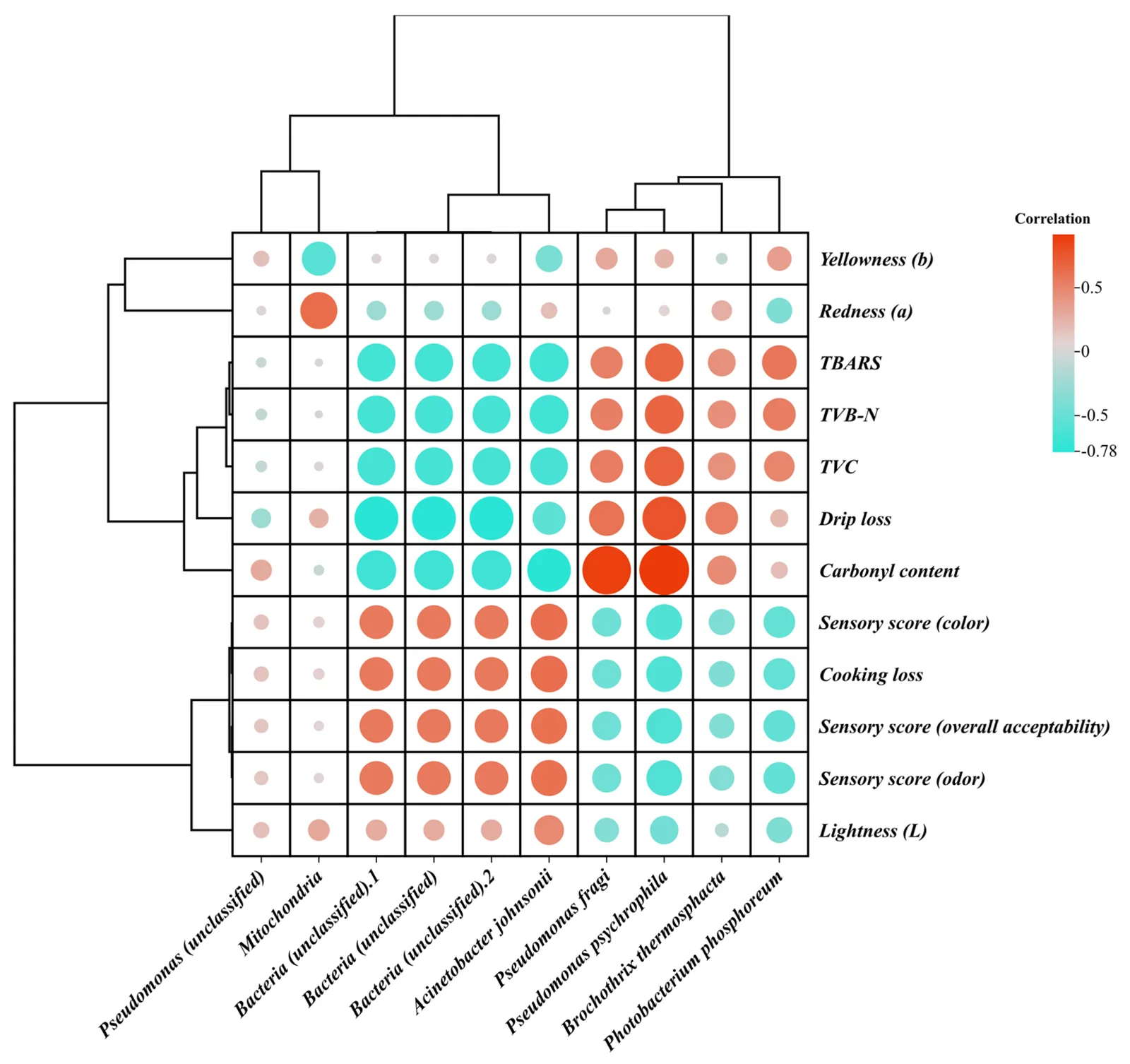
Spearman correlation heatmap showing associations between dominant bacterial genera and physicochemical and sensory indices across all samples (*n* = 21).

Figure 15 summarizes exploratory Spearman associations between dominant bacterial taxa and quality indices across 21 paired observations. Because taxon proportions and quality variables changed concurrently with storage time and treatment, the correlations may reflect shared temporal or treatment structure, compositional constraints, or other unmeasured factors. They do not establish directionality, mediation, or a direct causal mechanism. *Pseudomonas* relative abundance was positively associated with TVB-N, indicating that the two variables covaried within this dataset. Among the evaluated taxon-quality pairs, *Pseudomonas psychrophila* showed the strongest positive association with protein carbonyl content (Spearman’s ρ = 0.918, Benjamini–Hochberg-adjusted *p* = 5.47 × 10^−7^). This association is hypothesis-generating and does not demonstrate that the taxon produced nitrogenous compounds or caused protein oxidation, particularly because the sequencing data are compositional [42]. The lower endpoint TVB-N value and smaller *Pseudomonas* proportion under PS-NPs are therefore reported as parallel observations rather than a causal chain.

#### 3.4.2. Associations with Oxidative Indices

TBARS and protein carbonyls described oxidative stability in relation to microbial community changes. By day 24, CK TBARS had risen to 0.46 mg MDA/kg, while protein carbonyls reached 4.68 nmol/mg protein. The corresponding PS-NPs values were 36.96% and 19.44% lower than CK, respectively. *Pseudomonas fragi* and *Pseudomonas psychrophila* were positively associated with several deterioration indices and negatively associated with sensory scores Figure 15. These variables covaried, but the correlations do not identify causal pro-oxidative activity because storage time, treatment, microbial proliferation, and chemical oxidation were not separated in a causal model. One hypothesis is that a lower lipid-oxidation burden reduced lipid-derived radicals and aldehydes capable of promoting protein carbonyl formation [35,41,42,43]. An alternative, non-microbial explanation is a direct antioxidant effect of the active pad. Both explanations require targeted radical, aldehyde, and intervention measurements.

#### 3.4.3. Associations with Physical and Sensory Indices

Correlations involving *Pseudomonas*, *Brochothrix*, redness, water-holding capacity, and sensory scores are observational. By day 24, a* had declined to 4.26 in CK but remained at 8.08 under PS-NPs (Figure 15). A hypothesis raised by this pattern is that lower aerobic spoilage activity reduced oxygen depletion and slowed metmyoglobin formation; however, oxygen consumption and myoglobin redox state were not measured. Cooking loss was 32.18% in CK and 28.49% under PS-NPs. A second hypothesis is that lower proteolytic or oxidative damage preserved myofibrillar protein–water interactions and reduced heat-induced water release [41,42]. These pathways are proposed only as testable hypotheses. Direct antioxidant and moisture-management effects of the pad, as well as shared dependence on storage time, remain alternative explanations.

## 4. Conclusions

The nanoparticle-loaded PVA/SPI pad was associated with slower microbial and physicochemical deterioration of pork stored at −1 °C. Compared with CK and PS, PS-NPs delayed the increase in cultivable bacterial counts, TVB-N, TBARS, protein carbonyls, and cooking loss. It also retained greater redness and higher sensory scores. Sequencing showed treatment-related differences in community composition, including a lower *Pseudomonas* proportion under PS-NPs at day 24. Exploratory correlation analysis identified covariation between spoilage-associated taxa and nitrogenous, oxidative, water-holding, and sensory indices, but it did not establish causality. These results extend earlier performance-focused work by connecting active-pad treatment with concurrent microbial-community and quality trajectories under superchilling. The study is limited by three biological replicates per treatment-by-time combination and the inability of 16S relative abundance to establish absolute taxon inhibition. Accordingly, the proposed microbial, oxidative, color, and water-holding pathways are hypotheses requiring isolate-level, community-wide, and targeted chemical validation.

## Figures and Tables

**Table 1:** The L*, a* and b* value of fresh pork stored at different storage treatments.

	Experimental Groups	Storage Time (d)						
		0	4	8	12	16	20	24
L^*^	CK	54.50 ± 1.07 ^ab^	55.18 ± 1.46 ^a^	55.29 ± 0.46 ^a^	54.35 ± 1.18 ^ab^	52.66 ± 1.05 ^b^	50.39 ± 1.06 ^c^	50.16 ± 0.98 ^c^
	PS	54.50 ± 1.07 ^a^	54.88 ± 1.10 ^a^	54.50 ± 1.13 ^a^	53.51 ± 0.73 ^ab^	51.59 ± 0.84 ^b^	49.16 ± 0.97 ^c^	48.63 ± 0.91 ^c^
	PS-NPs	54.50 ± 1.07 ^a^	56.24 ± 0.56 ^a^	55.51 ± 1.00 ^a^	54.82 ± 1.31 ^a^	56.23 ± 1.29 ^a^	54.34 ± 1.14 ^ab^	52.23 ± 1.00 ^b^
a^*^	CK	6.74 ± 0.63 ^cd^	7.89 ± 1.14 ^c^	9.78 ± 0.99 ^a^	8.85 ± 0.81 ^ab^	7.34 ± 0.69 ^a^	5.55 ± 1.24 ^de^	4.26 ± 0.65 ^e^
	PS	6.74 ± 0.63 ^b^	7.49 ± 0.76 ^ab^	9.05 ± 0.88 ^a^	8.11 ± 0.67 ^ab^	6.52 ± 0.90 ^bc^	4.89 ± 1.05 ^cd^	4.88 ± 0.76 ^d^
	PS-NPs	6.74 ± 0.63 ^b^	8.09 ± 0.86 ^b^	10.40 ± 0.99 ^a^	10.89 ± 0.64 ^a^	9.96 ± 0.98 ^ab^	7.87 ± 1.19 ^ab^	8.08 ± 0.82 ^b^
b^*^	CK	14.50 ± 1.24 ^bc^	12.20 ± 1.21 ^d^	13.83 ± 0.69 ^bcd^	12.82 ± 0.49 ^cd^	15.60 ± 1.05 ^c^	15.19 ± 1.16 ^ab^	16.55 ± 0.77 ^a^
	PS	14.50 ± 1.24 ^abc^	12.63 ± 0.93 ^c^	13.81 ± 1.10 ^bc^	14.48 ± 0.80 ^abc^	15.84 ± 1.27 ^ab^	15.61 ± 0.89 ^ab^	16.20 ± 0.71 ^a^
	PS-NPs	14.50 ± 1.24 ^a^	11.32 ± 0.62 ^cd^	10.88 ± 1.12 ^d^	11.53 ± 0.44 ^cd^	12.71 ± 0.68 ^bc^	13.67 ± 1.05 ^ab^	14.90 ± 0.69 ^a^

Note: Values are expressed as mean ± standard deviation (SD). Different lowercase letters (a–e) within the same column indicate significant differences (*p* < 0.05).

## Data Availability

The original contributions presented in the study are included in the article; further inquiries can be directed to the corresponding authors.
